# Reviews of Books

**Published:** 1924-11

**Authors:** 


### REVIEWS OF BOOKS.

### DOCTOR AND SPORTSMAN.

**Reminiscences of an Old Physician.** By ROBERT BELL, M.D., F.R.F.P.S. Murray. 16s. net.

Dr. Bell had achieved many distinctions long before he came prominently before the lay public as a bold and convinced opponent of the surgical treatment of cancer. Naturally, his "Reminiscences" have much to say on that troubled subject—and something, too, upon a medical *cause célèbre* in which he obtained heavy damages for libel. Dr. Bell tells us that in 1910 he was offered a baronetcy which he declined for domestic reasons, and he expresses his conviction that had he accepted it the fact would have prevented the publication of the article which led to legal proceedings two years later. Since then he has had a difficulty with the General Medical Council, and he complains strongly of the attitude of that body towards him. Happily, all these things are in the past, and Dr. Bell is sturdily continuing his efforts to show that a medical man is not necessarily a "quack" because he differs from the majority of his brethren about the treatment of cancer or any other disease. Dr. Bell's views may be right or wrong—time and wide experience only can show—and all that need be said is that if there is one subject more than another upon which it is folly to dogmatise, it is cancer.

Dr. Bell's book is not written primarily *ad clerum*, and the "general reader" will find in it much that is of interest in the way of accounts of travel and sport, told with a rather engaging touch of garrulous complacency. He has been a sportsman from boyhood and an especially successful angler—the trout must have learned to dread the "off-days" which he managed to snatch from his busy life in Glasgow. His prowess began at a tender age—he was, apparently, about ten years old when he had a memorable day on the Aln, at Alnwick, where he was born. With one cast of flies he landed eleven dozen trout, weighing close upon forty-three pounds. "I never, either before or since, have seen trout rising so greedily to the fly—sometimes I was able to land two at a time, and all were fairly good-sized trout and in fine condition." Both elementary and secondary education among trout have made long strides since those halcyon days. Longer holidays were spent in travels of which Dr. Bell gives accounts that are often amusing if not very novel. Meanwhile, he was acquiring a high reputation as a gynæcologist and in other specialist directions, and he appears to have been the first to recognise that constipation is not only a prolific cause of disease, but a disease in itself. The layman as well as the doctor will read with curious interest much that he has to say of what were once new treatments of diphtheria and cancer, but both may, perhaps, be excused if they skip his verses. We prefer Dr. Bell, the physician, the angler and the anecdotist, to Dr. Bell, the poet. One of his anecdotes is rather poignant—it tells us of a consultant whose income fell from £3,000 a year to £300 when the Great War broke out.

With Dr. Bell's parting shot at the teetotallers we must leave him. He tells us frankly that it was only "free libations of good old port" which enabled him to stand the heavy strain of work during the busiest period of his life. "This so-called poison" has never injured his health one whit, but he thinks he would probably have been in his grave a generation ago had he lived under a *régime* of Prohibition. Unhappily, we are not all so robust, either in our bodies or our convictions, as this genial "old physician."

### "SLEEPY SICKNESS."

**Epidemic Encephalitis.** By ARTHUR J. HALL, M.D., F.R.C.P. Illustrated. (John Wright and Sons, Bristol. 12s. net.)

**Chronic Epidemic Encephalitis.** By AUGUST WIMMER, M.D. Illustrated. (Heinemann. 21s. net.)

Few diseases are shrouded in greater mystery than that which has recently come into prominence under the popular name of "sleepy sickness." *Encephalitis lethargica*, as it was called by Von Economo of Vienna, who published the first description of it in 1917, is strangely polymorphous in its salient features, and the lethargy which characterised the earlier outbreaks has not been so prominent in the later epidemics. Though at first regarded as a new clinical entity, and thought by some to be associated with certain food poisoning, research in medical literature has revealed a number of outbreaks of a similar character in past times, notably the *Schlafsucht* epidemic at Tubingen in 1712-3, and the Italian *Nona*.

The precise epidemiological characters of encephalitis have yet to be determined, though, as Crookshank, Hamer and others have shown, there appears to be some not fully understood association with epidemics of influenza. Interesting as are the clinical aspects of the infection, it is the sequelæ which give to the disease its chief importance and its most sinister features. The heightened susceptibility to emotional stress and the strange perversions of conduct whereby a previously well-behaved child becomes a persistent pilferer, or exhibits still more serious derelictions from the paths of decency and social order, raise questions of a psychological nature of a far-reaching character in relation to responsibility. If a child after an attack of encephalitis shows all those traits of conduct which have been attributed to the condition of so-called "moral imbecility," it is a reasonable assumption that similar behaviour in children who are not known to have so suffered may be due to comparable physical changes in the brain tissue.

These behaviour-phenomena supervene so constantly (they have been described in Austria, Denmark, England, Germany and the United States), and are so true to type whenever they occur, that they clearly form a symptom-complex characteristic of encephalitis. No subject of greater interest could have been chosen for the Lumleian Lectures, and Prof. Arthur Hall has made good use of his opportunity. He has discussed and arranged in logical order the amazing mass of material which has been published upon the subject and illustrated his lectures with a fine series of photographs and charts. How great is this amount of material may be gauged from the fact that, together with the bibliography included by Dr. Parsons in the Ministry of Health Report, he gives 2,064 references, which fill 74 pages of closely printed type.

Dr. August Wimmer treats the subject from a more strictly clinical standpoint, though he is less concerned with the clinical picture of acute encephalitis, but rather with its character as a chronic and progressive disease of the nervous system. As Professor of Psychiatry and Director of the Psychiatric Laboratory of the University of Copenhagen, and Chief of the clinic for nervous and mental disease, he has had unrivalled opportunities of meeting with the later manifestations of this infective process. It is especially in these later phases that the difficulty of diagnosis shows itself, for not infrequently the initial onset has not been severe, or its true nature has not been recognised. The author devotes considerable attention to the question of diagnosis and prognosis, and illustrates his conclusions by the citation of a large number of cases and photographic reproductions. A section is also devoted to the pathology and the results of experimental inoculations on rabbits carried out in the University Pathological Laboratory. Dr. Wimmer has a wide acquaintance with the Continental—and especially the French—literature on Encephalitis.

It is not a little gratifying that he should have chosen to write his book in English. In his preface he mentions the fact that he owes his intiation into English Neuropathology to his training at the National Hospital for the Paralysed, Queen's Square. He offers his work as " a modest tribute " to the memory of Sir Victor Horsley and Dr. Beevor. Though printed in Copenhagen the typographical errors are exceedingly few, and the reproductions of the photographs are excellent. The book is not only valuable in itself as a contribution to the literature of Encephalitis, but it is also a *pignus amicitiæ*—a witness of that close bond which unites the two countries.

### WITCHES AND DEMONS.

**King James the First's "Daemonologie," and "Newes from Scotland."** Edited by G. B. Harrison. (The Bodley Head Quartos: Lane. 3s. net.)

In King James the First's little volume, " Daemonologie," we find an epitome of the blatant futilities of which the " reasoning animal " is capable. In the form of a dialogue between Philomathes and Epistemon the King sets forth the generally accepted beliefs regarding witches and sorcerers, how they make compacts with the devil and how they make use of his assistance to obtain power or to overcome their enemies; in what form he appears to them and in what manner they show their adoration of him; and so on through many—and at times indecent—details. And, as usual, he supports it all, satisfactorily from his own point of view, by reference to the Scriptures.

King James was a man of no inconsiderable learning—was he not " the most learned fool in Christendom " ?—but he was credulous to a degree. He was, too, mightily exercised in his mind by the wave of rationalistic thought which had set in not long before and was exemplified in such books as the " De præstigiis daemonum," by Johann Wier, published in 1563, and " The Discoverie of Witchcraft," by Reginald Scot (1584). He inveighed against the " damnable opinions of Wierus and Scot," and had the " Discoverie " burnt by the hangman. The wonder is that he did not try to have the author burnt also. In the " Newes from Scotland " is recorded the appalling fate of a certain Dr. Frian who was accused of being a sorcerer. He was examined by the King himself and was subjected to the most hideous tortures—which are recorded here in gruesome detail. As he would not confess he was " put into a carte and beeing first strangled, hee was immediately put into a great fire, being readie provided for that purpose, and there burned in the Castle hill of Edenbrough on a saterdaie," in the year 1591. But by the time James had " commenced author " burning was not quite so easy for the King of England as it had been years before for the King of Scotland.

Although these writings have been frequently referred to by those who have dealt with the subject of witchcraft, they have for long been almost inaccessible. It is satisfactory, therefore, to see them now included in this admirable series. It is a pity that Scot's " Discoverie " is too long to be reprinted in a similar form.

### SURGICAL HANDICRAFT.

**Minor Surgery and Bandaging.** By Gwynne Williams, M.S., F.R.C.S. 18th Edition. (J. and A. Churchill. 10s. 6d. net.)

As the reclothed skeleton of the work originated by Christopher Heath in 1861, the present edition has, in the able hands of Mr. Gwynne Williams, been brought fully up to the requirements of the most modern surgical technique. The treatment of fractures forms no inconsiderable part of the work of house-surgeons and dressers, and the non-operative management of these injuries has been well revised. An account is given under this section of Delbet's method of applying plaster in fractures about the lower third of the leg. In view of possible litigation the author wisely recommends that a radiogram be taken in all cases of fracture before setting it permanently.

The subject of blood transfusion has come into prominence lately, and the method of performance in which sodium citrate is added to the blood to prevent coagulation is described. Some of the most difficult cases that come under the care of the house-surgeon are those of septic infection of the fingers and hands, owing to the grave risk of general infection as well as of permanent disability of the hand. In view of the recent work of Kavanel, explicit directions are given as to the opening up of the various tendon sheaths. A valuable chapter on anæsthetics, local, regional and spinal, is contributed by Drs. Dudley Buxton and Felix Rood, in which it is stated that the safest way to give chloroform is to employ a regulating inhaler, such as that known as the Vernon-Harcourt. All the principal minor surgical emergencies likely to be met with in hospital or in private practice are fully described, together with their appropriate treatment, in this classic little manual, which is not only indispensable to house-surgeons and dressers, but will also be found valuable for reference by all practitioners.

### REGULATING THE DOCTOR.

**Methods in Medicine.** By George R. Herrman, M.D., Ph.D. (Kimpton. 30s. net.)

In its conception this work is based upon the local rules and regulations of the Robert Abbott Barnes Hospital, St. Louis. It has developed into a heavy volume of 500 pages, of which 90 are devoted to representations of specimen charts for recording the results of investigation of the patients passing through the "service." It requires a genius for organisation and a congenital love for system and routine to produce such a work as this. Its value to the staff of its home hospital may be great, and ignorance or disobedience of its precepts doubtless entails drastic penalties, but its value to the medical world at large is marred by its bulk, its insistence upon local and unimportant details, and its scanty treatment of the subject of bedside examination.

There are chapters upon laboratory diagnosis and diet which are clearly written and are worthy of a better setting than they receive—they are lost in a mass of rules and regulations which remind the reader of the Army Act. The picture of a doctor locking himself into the record room at night, looking up the record (probably a very bulky file!), then locking himself out again and posting the key through the letter-box may raise a smile; it will not help him to treat his patient with increased skill. It is impossible to recommend the book to medical men trained on this side of the Atlantic.

### A COMPLETE COMPILATION.

**On the Breast.** By Duncan Fitzwilliams, C.M.G., M.D., Ch.M., F.R.C.S., Lecturer on Operative Surgery to St. Mary's Hospital. (Heinemann. 30s. net.)

To read a relatively large book entirely devoted to one small part of the human body, and then to find the whole subject still retaining its interest, reminds us very forcibly of the amazing complexity and breadth of all medical science. Still there is no doubt that, of all organs, the breast is one of the most inadequately treated in books upon general surgery, and the author has therefore done a signal service to the profession, not only by thoroughly and minutely reviewing our present knowledge, but by adding many valuable suggestions of his own. Perhaps the keynote of the work is the need, in cases concerning the breast, of the earliest possible diagnosis and the best means of making it. Being a region where the surgery of early conditions is almost as simple as the later surgery is difficult, there is abundant excuse for thus discussing all problems involving the mammary region. Something of an innovation occurs with the author's invitation to his readers to send details and specimens of any breast case of pathological or clinical interest which they would like investigated and recorded to him at St. Mary's Hospital.

The system upon which the book is compiled follows more or less orthodox lines. The normal development of the breast is first discussed; then the anatomy, normal and abnormal. Next come the various anomalies of lactation, the abscesses and suppurative or inflammatory conditions and wounds. The space devoted to tumours, their diagnosis and prognosis, is considerable, and advisedly so. Cancerous conditions are discussed both in their laboratory and clinical aspects, while the outlook under modern surgical procedures is fully described. A specially interesting chapter by Dr. Orton is devoted to X-ray treatment of malignant disease of the breast. The whole is a remarkably complete compilation, and one upon which the author is greatly to be congratulated.

### THE TREATMENT OF DISEASE.

**The Nature of Disease.** By J. E. R. McDonagh, F.R.C.S. Illustrated. (Heinemann. £3 3s.)

A book with so striking a title inevitably attracts attention, and in this large volume Mr. J. E. R. McDonagh, F.R.C.S., of the London Lock Hospital, has given us some most original conceptions. It is true that he devotes his chief attention to one contagious parasitic disease, but there is a good deal in his saying that if you study one disease in its entirety you study medicine. In this case, having dealt in the greatest detail with the pathological aspect of venereal infections and their after-effects on the body, he proceeds to suggest a host of interesting analogies and deductions which might be made in connection with the known course of other diseases.

It is impossible here to enter upon and illustrate the technical side of his conclusions, which will, indeed, not be accepted by everybody, but attention should be called to the work as exemplifying a new if somewhat reactionary line in medical research. Once break through a long accepted sequence of facts and theories, and on every hand all sorts of fascinating possibilities appear. In this respect, at least, we can indicate some of the author's hopes. It may, he thinks, be possible along the paths already opened to get a clearer view of the physical changes which the protein particles in the body undergo when they become cancerous. There is, he feels, a possibility that combining chemical with physical research upon the actions of the more chemically complex alkaloids will result in enormous simplification in the treatment of disease. These two developments alone would represent epoch-marking discoveries. We can only hope that his optimism will ultimately be justified. Certainly his suggestions are worthy of close reading.

### THE FATE OF THE REJUVENATED.

**Rejuvenation and the Prolongation of Human Efficiency.** By Dr. Paul Kammerer. (Methuen. 8s. 6d.)

**The Theory and Practice of the Steinach Operation.** By Dr. Peter Schmidt. (Heinemann. 7s. 6d. net.)

Some two years ago Dr. Kammerer published a German book on the subject of rejuvenation, but the present volume is not merely a translation, but practically a new book which gives an account of the work of the past two years as well as of the investigations which led up to it. The German book contained illustrations of animal experiments. The present one contains photographs of the human subjects of rejuvenation experiments, and in this respect, as in many others, we find that great advances have been made during the last few years. The author, who is an assistant of Steinach's, and his ardent admirer, attempts to give, in popular phraseology, an account of the theories as well as of the practical results of the Steinach school. Here and there he is unnecessarily florid, but that is a minor matter. He shows that rejuvenation is feasible for women as well as for men, and that it can be brought about by such apparently unrelated processes as the ligature of certain ducts, the transplantation of certain glands and exposures to the X-rays.

With regard to the future, he is careful not to hold out too rosy prospects. Rejuvenation, he says, cannot make immortals. For which assurance we cannot be too thankful—who among us is there willing to follow in the steps of the Wandering Jew? The author assures us, however, that the Steinach processes distribute the various stages of life differently. The prime of life is extended at the cost of that phase in our existence when our juniors wonder why we do not retire and make place for them. When senility does ultimately overtake rejuvenated man, it is not a sneaking, chronic disease, but an acute sickness, the decline being the more rapid because it has been postponed. This may be a consolatory doctrine to the temperament that prefers taking a "header" into the unknown instead of wading gradually into it. But there is something eerie about the fate of the man the candle of whose life is made to burn with artificial brightness till the last moment, when—puff! and it is extinguished.

"The Theory and Practice of the Steinach Oper ation" is also a translation from the German. Dr. Schmidt is a co-worker with Professor Steinach, and his book consists to a great extent of reports of one hundred cases in which the operation has been performed. A useful introduction is added by Dr. J. Johnston Abraham, C.B.E., D.S.O., who points out that these operations have nothing whatever in common with those of Voronoff on monkey glands, although the two are often confused, even by medical men. Dr. Johnson thinks that, hitherto, too much stress has been laid upon the sexual effects of the Steinach operation and too little upon the more important increase in mental alertness and physical endurance.

### MISCELLANEOUS NOTICES.

### The Diabetic Diner.

Food for the Diabetic. What to Eat and How to Calculate it with Common Household Measures. By M. P. Huddleson. (Macmillan Co. 6s. net.)—This book is written essentially for the patients themselves, and gives briefly and in simple language most useful information of the nature of their disease from a dietetic standpoint. Very usefully, too, the functions of food in the normal individual are described, as well as those in the diabetic, since it is impossible for the patient to understand his treatment unless he appreciates the difference between normal standards and his own. The quantities mentioned are given in grams, in ounces and in common household measurements, so that all can be satisfied. From "Rules to be Observed by the Diabetic Patient" down to "Menu-Planning" this is a thoroughly useful little book.

### A Manual on the Mind.

The Mind and How to Manage it. By Psychologist. (Mills and Boon. 3s. 6d. net.)—Those who wish to know something of the workings of the mind and how to keep it in a healthy condition will welcome this little manual. It is written in a simple straightforward way, and will enlighten the reader and not confuse him as so many of these books are inclined to do. "Psychologist" writes on such matters as The Mechanism of the Mind, Symptoms of Nervousness, Fatigue, Memory and Mental Healing, and he is always brief and to the point, though of course intentionally elementary.

### For Youthful Students.

British Red Cross Society Junior Health Manual. No. 3. By Beatrice Agar. (Cassell. 1s. 6d. net.)—This is a capital little book for boys and girls, presenting facts about health in a bright and sensible way. But we are sure that any modern girl would take exception to the suggestion that a nurse's uniform is an ideal garment possessing "no fussy odds and ends." Accustomed to a frock slipping over the head and hanging from the shoulder, the nurse's dress fastening round the waist, and with high collar and cuffs, appears to most girls a rather unendurable garment. And Miss Agar must surely know that "fussy odds and ends" are not at the present in fashion.

### Dr. Hall's Essays and Addresses.

Essays and Addresses on Digestive and Nervous Diseases and on Addison's Anæmia and Asthma. By Arthur F. Hurst, M.A., M.D.Oxon, F.R.C.P., Physician, Neurologist and Director of Advanced Studies, Guy's Hospital. Illustrated. (Heinemann. 21s. net.)—In this volume of 320 pages are collected contributions that Dr. Hurst has on various recent occasions made to medical literature. In their collected form they contain, however, new matter and are the latest product of an original and elastic mind. Each essay is a model of convincing presentation of its subject and is pregnant with original thought and acute observation. Discussion of nervous and abdominal troubles forms the larger part of the volume, but there is a remarkably interesting essay on Addison's Anæmia to the alternative description of which as "pernicious" the author takes a not unnatural exception. The book may be recommended with confidence to the practitioner. He will find therein much that is new, much that is stimulating and much that will give him real help in dealing with some of the very difficult types of cases with which all doctors are at times confronted.

### Oto-Laryngology in General Practice.

Ear, Nose and Throat Treatment in General Practice. By Georges Portmann, M.D. Translated and edited by R. Scott Stevenson, M.D. (Heinemann. 10s. 6d. net.)—Text-books upon diseases of the ear, nose and throat, even when written for students, are not always as full in the sections devoted to treatment as might be desired. The original French edition of Dr. Portmann's work has already been received with favour, and Dr. Scott Stevenson is to be congratulated upon a translation which renders a book which specialises in treatment more readily available to his English speaking colleagues. The various morbid conditions treated of are only summarily described, but the wealth of prescriptions for all sorts and conditions of the different diseases will be greatly welcomed by general practitioners. There are several illustrations of apparatus used for diagnosis and treatment, among which may be noted the simple form of funnel employed by Moure and Lavielle for direct intra-laryngeal heliotherapy. An easy method of intra-laryngeal medication by means of a hypodermic syringe, the solution being injected directly through the skin, is novel and can be undertaken by any practitioner who is not accustomed to laryngoscopic work. The book will be a valuable addition to the practitioner's library.

### A New Philosophy.

Shaping a New Philosophy. By F. Attfield Fawkes. (Simpkin Marshall. 2s. net.)—This little book deals with what its author describes as "tool philosophy." Man has at his disposal enormous numbers of tools "of every conceivable size, form and character in order to shape his world of life. What he makes of that world depends entirely upon what tools he employs and how he uses them. To choose only the best and to use these wisely and well is man's chief work." Tools there are of the mind as well as the tools made by the hand. Mr. Fawkes develops his theories in an original way and there are many odd little items of information in the book. We learn, for instance, that there are 1918 references to love in Shakespeare, that there is in existence a little machine which changes £1 and 10s. notes into silver, and that in the 'nineties tram drivers and conductors received 4s. and 3s. respectively for a seventeen-hour day. There are chapters on Health Tools and on Crime Creative Tools, which may be of particular interest to our readers.

